# Novel insight into the regulatory roles of diverse RNA modifications: Re-defining the bridge between transcription and translation

**DOI:** 10.1186/s12943-020-01194-6

**Published:** 2020-04-17

**Authors:** Hanhan Shi, Peiwei Chai, Renbing Jia, Xianqun Fan

**Affiliations:** 1 Department of Ophthalmology, Ninth People’s Hospital, Shanghai JiaoTong University School of Medicine, Shanghai, 20025 P.R. China; 2 Shanghai Key Laboratory of Orbital Diseases and Ocular Oncology, Shanghai, 20025 People’s Republic of China

**Keywords:** RNA modifications, m^6^A, m^1^A, m^5^C, diseases

## Abstract

RNA modifications can be added or removed by a variety of enzymes that catalyse the necessary reactions, and these modifications play roles in essential molecular mechanisms. The prevalent modifications on mRNA include N6-methyladenosine (m^6^A), N1-methyladenosine (m^1^A), 5-methylcytosine (m^5^C), 5-hydroxymethylcytosine (hm^5^C), pseudouridine (Ψ), inosine (I), uridine (U) and ribosemethylation (2’-O-Me). Most of these modifications contribute to pre-mRNA splicing, nuclear export, transcript stability and translation initiation in eukaryotic cells. By participating in various physiological processes, RNA modifications also have regulatory roles in the pathogenesis of tumour and non-tumour diseases. We discussed the physiological roles of RNA modifications and associated these roles with disease pathogenesis. Functioning as the bridge between transcription and translation, RNA modifications are vital for the progression of numerous diseases and can even regulate the fate of cancer cells.

## Introduction

**Fig. 1:**
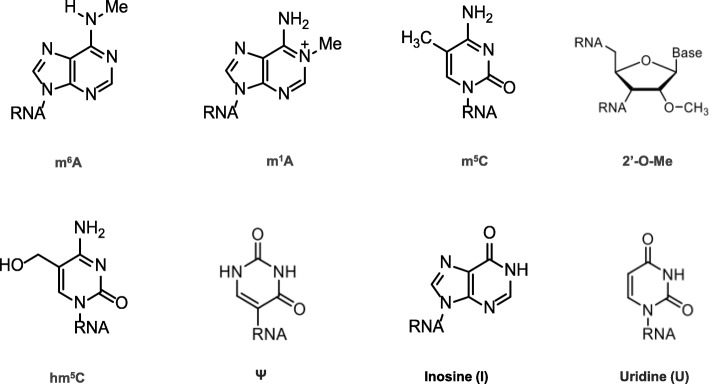
Chemical structures of mRNA modifications. Chemical structures in eukaryotic mRNA including m^6^A, m^1^A, m^5^C, hm^5^C, Ψ, I, U and 2’-O-Me

**Fig. 2:**
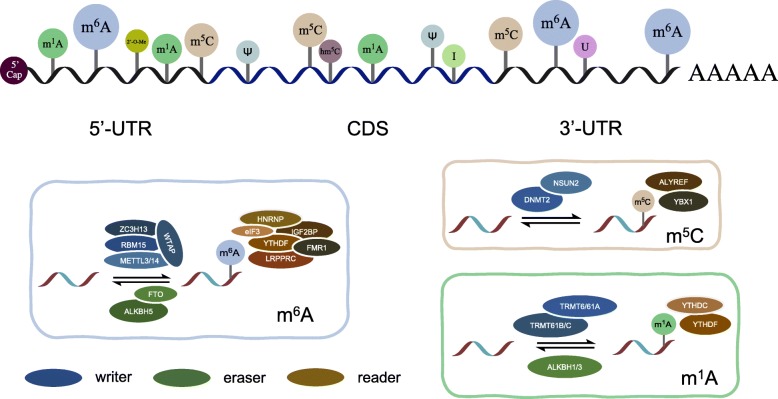
Locations of chemical modifications in mRNA. Chemical RNA modifications are shown in mRNA with their approximate distribution in transcripts. m^6^A with a widespread distribution prefers to be located in the consensus motif in the 3’UTRs as well as the 5’UTRs, which closely correlate with translation. Although m^1^A-containing mRNA is 10 times less common than m^6^A-containing mRNA, m^1^A is discovered in every segment of mRNA, including the 5’UTRs, CDS and 3’UTRs and mostly in highly structured 5’UTRs. Analogous to m^1^A, m^5^C can occur in coding and non-coding regions of mRNA, especially in GC-rich regions. Nevertheless, m^5^C within different positions regulates transcription differently. Tet-family enzymes prefer to oxidize m^5^C modifications in coding regions, so hm^5^C has a greater possibility of being present in CDS. Subsequently, Ψ is demonstrated to have a diversified location, whereas I is present at a large number of sites in the CDS, and U accumulates in 3’UTRs. 2’-O-Me focuses on decorating specific regions of mRNA that encode given amino acids. Additionally, as reversible modifications, most have their own readers, writers and erasers

In the 1950s, the first RNA nucleoside modification was identified [1]; since then, researchers have focused on updating the understanding of RNA modifications. At the very beginning, the 5’cap and the poly(A) tail, which represent cap and tail modifications, respectively, were discovered. However, with the limitations of technology, modifications of eukaryotic mRNA ends were considered the only post-transcriptional alterations to mRNA for a while. Fortunately, this situation did not last for a long time. Internal mRNA modifications have been investigated in succession in the last 50 years. The revealed mRNA modifications included but were not limited to N6-methyladenosine (m^6^A), N1-methyladenosine (m^1^A), 5-methylcytosine (m^5^C), 5-hydroxymethylcytosine (hm^5^C), pseudouridine (Ψ), inosine (I), uridine (U) and ribose-methylation (2’-O-Me) [2–4] (Figs. 1 and 2). m^6^A is the most abundant modification and was therefore thoroughly investigated [5].

Analogous to mRNA modification, we also identified many modifications on transfer RNAs (tRNAs) and ribosomal RNAs (rRNAs), such as queuosine (Q) [6]. Eukaryotic tRNAs contain, on average, over 10 modifications per molecule. From elementary isomerization or methylation to complicated modifications of ring structures, the number of tRNA modifications is the largest and has the widest chemical variety. Moreover, there are over 200 modifications on human rRNAs. Thus, their less complicated nature and greater abundance led to more investigations of tRNAs and rRNAs, even beyond mRNAs. Early studies have demonstrated that this variety of modifications leads to extra cellular functions for diverse RNA species [7].

### The regulatory role of RNA modifications

Modifications on different RNAs were found to regulate various cellular processes. Researchers demonstrated that these modifications can initiate translation, stabilize transcripts, splice pre-mRNA, facilitate nuclear export, etc. [8–12]. With respect to RNA modifications and technological advances in high-throughput sequencing and mass spectrometry, the mechanisms of different cellular processes influenced by RNA modifications are underexplored, including the less ubiquitous modifications on rare RNA species. tRNAs, which have the greatest number of types of different chemical modifications, regulate molecular mechanisms by selecting and protecting the reading framework, promoting tRNA decoding capability as well as changing codon-anti-codon connections [13–17]. Moreover, the functions of 2’-O-Me, Ψ and m^5^C, which are abundant in rRNA, have been investigated in detail. Without any doubt, mRNA modifications play roles in modulating molecular mechanisms. Subsequently, RNA modifications contribute to tumorigenesis by regulating cell survival, differentiation, migration and drug resistance [18].

### m^6^A RNA modification

#### Introduction to m^6^A RNA modification

m^6^A accounts for approximately 0.2~0.6% of total adenosines in mammalian RNA [2, 5]. General m^6^A modifications occur in mammals, plants, bacteria and even other types of eukaryotic RNA [19–22]. In addition to their widespread distribution, there is no less than 1-2 methylated adenosines in every single mRNA [23]. Studies have reported that m^6^A is located in the 3’ untranslated region (3’UTR), predominantly in a consensus motif, GGm^6^ACU [24–26]. Recently, m^6^A was also found in the 5’ untranslated region (5’UTR), a region that closely correlates with translation. It has been reported that methylated adenosine in the 5’UTR of mRNA can support cap-independent translation commencement and can increase translation [27, 28].

As a reversible mRNA modification, m^6^A has its own writers, readers and erasers. Methyltransferase-like 3 (METTL3) was the first demonstrated m^6^A writer [29]. In addition to METTL3, other proteins possessing methyltransferase (MTase) capability were recently identified, including methyltransferase-like 14 (METTL14), Wilms tumour 1-associated protein (WTAP), RNA-binding motif protein 15 (RBM15), KIAA 1429 and zinc finger CCCH-type containing 13 (ZC3H13) [30–33]. By binding to mRNA, readers, such as members of the YT521-B homology (YTH) domain family of proteins (YTHDF1, YTHDF2, YTHDF3, YTHDC1 and YTHDC2) and heterogeneous nuclear ribonucleoprotein (HNRNP) proteins (HNRNPA2B1 and HNRNPC) can execute the physiological functions of the modification [8, 10, 12, 34–38]. Additionally, eukaryotic initiation factor 3 (eIF3), insulin-like growth factor 2 mRNA-binding proteins (IGF2BP1, IGF2BP2 and IGF2BP3), fragile X mental retardation 1 (FMR1) and leucine-rich pentatricopeptide repeat-containing (LRPPRC) all can read m^6^A modifications [39, 40]. Both fat mass and obesity-associated protein (FTO) and alkB homologue 5 (ALKBH5) are erasers of m^6^A modifications [11, 41, 42].

#### Regulatory role of m^6^A RNA modification in molecular functions

Accumulation of pre-mRNA and diminution of mature mRNA in cyclo-leucine-treated avian sarcoma virus-infected cells and neplanocin A (NPC)-treated SV40 RNA demonstrate that m^6^A is essential in pre-mRNA splicing [43, 44]. Both cyclo-leucine and NPC are inhibitors of methylation that can be used to investigate m^6^A [45, 46]. Subsequently, MTases and demethylases might be involved in regulating RNA splicing. By changing RNA structure and regulating the combination of RNA and reader proteins, HNRNPC can modulate the splicing of m^6^A-containing mRNAs [10]. More recently, by relying on the RGG region in the low-complication region of HNRNPG, a reader was reported to cooperate with modified pre-mRNA and the phosphorylated C-terminal domain of RNA polymerase II to modulate splicing [47]. Moreover, FTO is vital to mRNA splicing because it prefers to bind to introns of nascent mRNA [48]. Another splicing-related eraser is ALKBH5. Immunofluorescence analysis revealed that ALKBH5 was tightly related to splicing factors [11].

Writers, readers and erasers can all regulate mRNA export. By modulating the clock genes Per2 and Arntl, METTL3 regulates the export of mature mRNA [49]. By interacting with SRSF3 and regulating the combination of SRSF3 and NXF1 on RNA, YTHDC1 mediates the export of modified mRNA [50]. Subsequently, knockdown of ALKBH5 leads to acceleration of mRNA export, suggesting that m^6^A is essential to regulating mRNA export [11].

AU-rich element (ARE), iron-responsive element (IRE) and cytoplasmic polyadenylation element (CPE) represent functional domains and are responsible for mRNA decay in 3’UTRs [51]. Coincidentally, m^6^A accumulates in 3’UTRs. Thus, the neighbouring sites of m^6^A and Hu antigen R (HuR), which is supposed to bind ARE to increase the stability of mRNA, lead to weak HuR function and mRNA instability [52]. However, ELAV1/HuR, a potential m^6^A-binding protein, can stabilize transcripts with the cooperation of the ARE domain [53]. Subsequently, it was reported that the stability of mRNA was decreased slightly in cells lacking ALKBH5 [11].

**Fig. 3:**
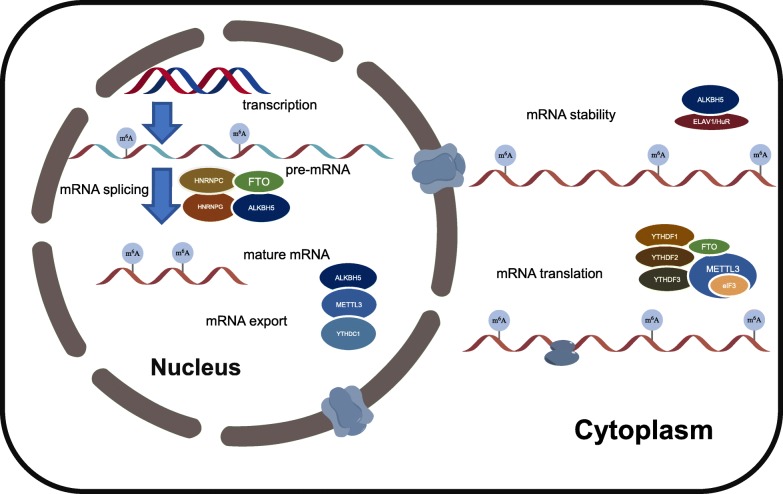
m^6^A RNA modification regulates physiological processes in cell. m^6^A RNA modification in mRNA plays an essential role in cellular processes, including mRNA splicing, mRNA export, mRNA stability and mRNA translation. Both readers (HNRNPC and HNRNPG) and erasers (FTO and ALKBH5) can modulate the splicing of mRNA. After splicing and combination, pre-mRNA evolves into mature mRNA. Regulated by ALKBH5, METTL3 and YTHDC1, mature mRNA is exported from the nucleus to the cytoplasm. Once exported to the cytoplasm, both ALKBH5 and ELAV1/HuR can maintain mRNA stability. Finally, numerous enzymes contribute to the process of translation. YTHDF1, YTHDF2, YTHDF3, FTO and METTL3 together with eIF3 can regulate translation with different mechanisms individually

The YTH domain family of proteins has a conserved m^6^A-binding pocket so that these proteins can tightly bind to m^6^A in a consensus sequence and directly transcribe the molecule [12, 26, 34–38]. Specifically, YTHDF2 accelerates mRNA decay by transferring RNA from the translatable pool to processing bodies [12]. Under heat shock conditions, dysfunction of FTO in 5’UTRs, which is regulated by YTHDF2, contributes to the promotion of cap-independent translation [28]. Moreover, YTHDF1 can increase the efficiency of translation by binding m^6^A [37]. Subsequently, YTHDF3 can regulate translation by both interacting with ribosomal proteins with bound YTHDF1 and by decaying the translation-related mRNA region with bound YTHDF2 [54, 55]. However, METTL3 can regulate translation flexibly because it can either recruit eIF3 to the initiation complex directly to increase translation or can inhibit translation efficiency [56, 57]. The translation efficiency is increased when METTL3 is knocked out in mouse embryonic stem cells (mESCs) and embryoid bodies (EBs) [57] (Fig. 3).

### m^1^A RNA modification

#### Introduction of m^1^A RNA modification

m^6^A has been reported to occur in DNA from a minor cluster of microorganisms and in RNA from an extensive range of organisms, and additionally, m^1^A was identified in the 1960s [58]. Rather than accumulating in mRNA, m^1^A is predominant in tRNA and rRNA, but we recently determined that it also exists in mRNA [59, 60]. However, m^1^A-containing mRNA is 10 times less common than m^6^A-containing mRNA [61, 62]. In tRNA and rRNA, m^1^A conserves the tertiary structure and affects translation [63, 64]. In mRNA, m^1^A has been discovered in every mRNA segment, including the coding sequence (CDS), 5’UTR and 3’UTR, although it is mostly found in the highly structured 5’UTR [62]. As a result, the location of the m^1^A methylated atom determines the function and mechanism of this kind of modification.

Because the distribution of m^1^A is imbalanced, the large number of m^1^A modifications on tRNA results in more tRNA m^1^A MTases than writers on mRNA. However, TRMT6/61A recognized a T-loop-like structure with a GUUCRA tRNA-like motif in mRNAs and decorated it with the m^1^A modification, TRMT61B installed m^1^A in mt-mRNA transcripts, and TRMT10C methylated the 1374 position of ND5 mt-mRNA [65, 66]. All of these phenomena contribute to tRNA m^1^A MTases and can function as mRNA writers. By binding to m^1^A-bearing RNA, YTHDF1, YTHDF2, YTHDF3 and YTHDC1 act as readers [67]. Subsequently, similar to ALKBH5 functioning as an eraser for m^6^A, ALKBH1 and ALKBH3 were able to demethylate m^1^A mRNA modifications [62, 68].

#### Regulatory role of the m^1^A RNA modification in molecular functions

It has been reported that m^1^A methylation occurs in highly structured or GC-rich regions of 5’UTRs (which is also the most frequent location) and may modify the predicted secondary structure, which hints at the potential of m^1^A to alter mRNA structural stability [61, 62].Moreover, m^1^A methylation can not only increase translation by decreasing the binding of the releasing factor but also prevent effective translation of m^1^A-containing CDS in mt-mRNA [26, 65]. Ultimately, it has been reported that the protein level is higher when a transcript carries the m^1^A modification around the initiation codon [69].

### m^5^C RNA modification

#### Introduction of the m^5^C RNA modification

m^5^C is a long-standing DNA modification that is essential for gene expression and epigenetic regulation [70, 71]. However, it can also be found in RNA. Although the m^5^C RNA modification can appear in both coding and non-coding regions, it has been reported to accumulate in the UTRs of mRNA and especially prefers to be located in GC-rich regions [72]. Since a number of studies have investigated the function of m^5^C in specific mRNAs, we concluded that m^5^C modifications in different locations (5’UTRs, 3’UTRs, coding regions) exert different transcriptional regulation activities [73].

It was revealed that m^5^C RNA modifications are catalysed by the NOL1/NOP2/SUN domain (NSUN) family of proteins (NSUN1, NSUN2, NSUN3, NSUN4, NSUN5, NSUN6 and NSUN7) as well as the DNA methyltransferase (DNMT) homologue DNMT2 [74–76]. However, among such diversified writers, only NSUN2 can install m^5^C on mRNA because rest of these proteins are writers of tRNAs and rRNAs. Subsequently, Aly/REF export factor (ALYREF), a specific mRNA m^5^C-binding protein that can read modifications, was identified as a reader of m^5^C [77]. According to liquid chromatography-tandem mass spectrometry analysis, YBX1 was defined as the other m^5^C reader that can maintain the stability of target mRNA [78]. Knowledge is limited about the protein factors responsible for removing modifications (Table 1).

**Table 1:** Writers, readers and erasers of the predominant mRNA modifications

RNA modification	Writers	Readers	Erasers
m^6^A	METTL3; METTL14; WTAP; RBM15; ZC3H13	YTH domain family of proteins (YTHDF1, YTHDF2, YTHDF3, YTHDC1 and YTHDC2); HNRNP (HNRNPA2B1 and HNRNPC); eIF3; IGF2BP (IGF2BP1, IGF2BP2, and IGF2BP3); FMR1; LRPPRC	FTO; ALKBH5
m^1^A	TRMT6/61A; TRMT61B; TRMT10C	YTHDF1; YTHDF2; YTHDF3; YTHDC1	ALKBH1; ALKBH3
m^5^C	NSUN2; DNMT2	ALYREF; YBX1	N.A.

#### Regulatory role of the m^5^C RNA modification in molecular functions

ALYREF, the reader of m^5^C, can adjust the export of transcripts by recognizing a unique RNA-binding motif [77]. Subsequently, NSUN2 adds m^5^C to both p27 mRNA at cytosine C64 in the 5’UTR and p21 mRNA in the 3’UTR [79, 80]. Deleting NSUN2 in human diploid fibroblasts (HDFs) can induce the elevation of p27, and overexpressing NSUN2 results in contrasting outcomes [79]. These results suggest that the m^5^C catalysed by NSUN2 in the 5’UTRs can limit the translation of p27. However, the m^5^C modifications added by NSUN2 to the 3’UTRs of p21 mRNA coordinate with the m^6^A modifications added by METTL3/METTL14 together to enhance the expression of p21 [80]. With regard to m^5^C modification in mRNA coding regions, it was revealed that in both bacterial whole-cell extracts and HeLa cell extracts, m^5^C could diminish translation significantly [27, 81]. Moreover, we demonstrated that when the m^5^C modification was present on interleukin-17A (IL-17A) mRNA, this modification could promote the translation of IL-17A [82]. The results of the above investigations revealed that the m^5^C RNA modification affects the expression of proteins by regulating both translation efficiency and transcript export (Table 2).

**Table 2:** mRNA modifications regulate the physiological process from transcription to translation

Modifications		Process	Enzymes involved	Description	Ref
m^6^A RNA modification		mRNA splicing	HNRNPC	HNRNPC modulates the splicing of mRNAs by changing RNA structure and regulating the combination of RNA and reader	[10]
			HNRNPG	HNRNPG cooperates with modified pre-mRNA and the phosphorylated C-terminal domain of RNA polymerase II to regulate splicing	[47]
			FTO	FTO prefers to bind to introns of nascent mRNA	[48]
			ALKBH5	ALKBH5 relates to splicing factors tightly according to the analysis of immunofluorescence	[11]
		mRNA export	METTL3	METTL3 regulates the export of mature mRNA by modulating clock genes Per2 and Arntl	[49]
			YTHDC1	YTHDC1 mediates the export of decorated mRNA by interacting with SRSF3 and regulating the combination of SRSF3 an NXF1 on RNA	[50]
			ALKBH5	Knockdown of ALKBH5 leads to acceleration in mRNA export	[11]
		mRNA stability	ALKBH5	The stability of mRNA was decreased slightly in RNA lacking ALKBH5	[11]
			N.A.	Neighbouring sites of m^6^A and HuR weaken the function of HuR and increase the instability of mRNA	[52]
			N.A.	ELAV1/HuR, which is one of m^6^A-binding proteins and stabilizes transcripts with the cooperation of the ARE domain	[53]
		mRNA translation	YTHDF2	YTHDF2 regulates translation by transferring the bound RNA from the translatable pool to processing bodies to promote mRNA decay	[12]
				YTHDF2 induces the dysfunction of FTO in the 5'UTRs and contribute to promoting cap-independent translation	[28]
			YTHDF1	YTHDF1 increases the efficiency of translation by binding to m^6^A	[37]
			YTHDF3	YTHDF3 interacts with ribosomal proteins along with YTHDF1 to regulate translation	[54]
				YTHDF3 decays of convinced translation related region in mRNA together with YTHDF2	[55]
			METTL3	When knocking out METTL3 in mESCs and Ebs, the translation efficiency is increased	[57]
				METTL3 recruits eIF3 to the initiation complex directly and enhance translation level	[56]
m^1^A RNA modification		mRNA stability	N.A.	m^1^A in highly structured or GC-rich regions of 5'UTRs alters mRNA structural stability by modifying the predicted secondary structure	[61, 62]
		mRNA translation	N.A.	m^1^A upregulated translation by depressing binding of releasing factor	[26]
			N.A.	m^1^A prevents effective translation of CDS in mt-mRNA	[65]
			N.A.	The protein level would be superior when the transcript was modified by m^1^A at/around the initiation codon	[69]
m^5^C RNA modification		mRNA export	ALYREF	ALYREF adjusts the export of transcripts by recognizing the unique RNA-binding motif	[77]
		mRNA translation	NSUN2	Deleting NSUN2 in HDFs can induce the elevation of p27, and overexpressing NSUN2 induces the opposite outcome	[79]
				m^5^C catalysed by NSUN2 in 3'UTRs of p21 mRNA coordinates with m^6^A methylated by METTL3/METTL14 together to enhance p21 expression	[80]
			N.A.	Translation diminishes significantly in both bacterial whole-cell extracts and HeLa cell extracts when m^5^C modifies the coding regions of mRNA	[27, 81]
			N.A.	m^5^C found on IL-17A mRNA can promote the translation of IL-17A	[82]
Other	hm^5^C	mRNA translation	N.A.	hm^5^C associates with translation activation in Drosophila	[69]
	Ψ	mRNA splicing	N.A.	Ψ, which is near the 3' splice site in the polypyrimidine tract, prevents pre-mRNA splicing by regulating U2AF	[83]
		mRNA stability	N.A.	The higher expression of heat shock-induced Pus7-dependent pseudouridylated transcripts in wild-type yeast than in Pus7-knockdown yeast indicates that Ψ has the capability to maintain stability of RNA	[84]
		mRNA translation	N.A.	Compared to U modifications located at similar sequences, Ψ-containing mRNA indicates an increase in translation levels of approximately 25%	[84]
			N.A.	Ψ doubles the expression of an unmodified transcript	[85]
			N.A.	When a separate Ψ modifies the special position of codon "UUU", mRNA translation can be limited	[81]
	I	mRNA structure	N.A.	I fastens pairs of nucleotides to influence the native secondary structure of mRNA	[86]
		mRNA translation	N.A.	Guanosine, adenosine and uracil are the products decoded from I by the translation machinery	[87]
	U	Protein expression	N.A.	Protein level alterations accompany C-to-U editing of RNA	[88]
	2'-O-Me	Viral RNA infection	N.A.	2'-O-Me-modified viral RNA disrupts native host antiviral responses by escaping suppression mediated by IFIT	[89]
		mRNA translation	N.A.	2'-O-Me modifies specific regions of mRNA that are translated to glutamate, lysine and glutamine, hinting that 2'-O-Me has the potential to affect translation efficiency	[90]

### Other RNA modifications

#### hm^5^C

m^5^C can be oxidized into hm^5^C via the function of the Tet-family enzymes [91–93]. Moreover, hMeRIP-seq showed that Tet-family enzymes prefer to oxidize m^5^C modifications in coding regions; these results indicate that hm^5^C is highly likely to be located in the introns and exons of coding transcripts. However, in contrast to m^5^C methylation in the coding regions of mRNA, which plays a negative role in translation, hm^5^C tends to associate with translation activation in Drosophila [69].

#### Ψ

As hm^5^C is analogous to the oxidization of m^5^C, Ψ is produced by the isomerization of U. Ψ is the most abundant RNA modification and prefers to accumulate in tRNA and rRNA; however, it has also been reported to be present on mRNA and snRNA [94, 95]. Interestingly, the number of Ψ sites in mRNA ranges from 96 to 2084 in humans [84, 96–98].

However, by regulating U2 auxiliary factor (U2AF), Ψ, which is near the 3’ splice site in the polypyrimidine tract, prevents pre-mRNA splicing [83]. Expression of heat shock-induced Pus7-dependent pseudouridylated transcripts is higher in wild-type yeast than in Pus7-knockdown yeast and indicates that Ψ has the capability to maintain RNA stability [84]. Nevertheless, modifications were examined at similar sequences, and compared to U-containing mRNA, Ψ-containing mRNA experienced an increase in translation by approximately 25% [84]. Such modifications could double the expression of translation when compared to blank control transcript without any modification [85]. Although Ψ can promote translation and enhance the lifespan of RNA, it has negative effects on protein expression [85]. It has been reported that Ψ-containing mRNA exhibits a 30% decrease in protein expression. Specifically, bacterial mRNA translation can be limited when a separate Ψ modification is present at a given position of codon “UUU”, especially at the third codon position [81]. Moreover, both in vitro and in vivo, the Ψ modification might change the nonsense codons into sense codons [99, 100]. Above all, some of these investigations were conducted by Ψ in artificial mRNA, and the function of Ψ in biological mRNA has yet to be elucidated.

#### I and U

Catalysed by adenosine or cytidine deaminating enzymes, RNA editing is a kind of programmed alteration [101]. However, rather than permanent DNA mutations or reversible RNA modifications, RNA editing has its own limited lifespan and results in more permanent modification [102].

Adenosine-to-inosine RNA editing (A-to-I editing), also called I, is catalysed by adenosine deaminases acting on RNA (ADARs) [101, 103, 104]. Recently, 1741 I sites have been reported in CD regions of transcripts from RNA-seq data of different human tissues [105]. Moreover, it has been reported that ADAR1 and ADAR2 act only on double-stranded regions, which limits the areas of mRNA that I can modify [106]. I can fasten pairs of nucleotides; thus, this widespread modification in metazoan mRNA can influence the native secondary structure of mRNA [86]. An in vitro translation system was implemented to scientifically test the decoding of I, revealing that guanosine, adenosine and uracil are the products decoded from I by translation machinery [87].

However, with regard to cytidine-to-uridine RNA editing (C-to-U editing), also called U, it has been reported that U accumulates in 3’UTRs, and over 70 new sites have been discovered by transcriptome-wide research [88, 107]. Subsequently, after exploring several intestinal mRNAs, it was revealed that the protein level is altered by C-to-U editing of RNA [88]. However, there is little research on the relationship between the expression of transcripts and U. The biological influence of U has yet to be investigated.

#### 2’-O-Me

Unlike how I and U are modifications on a base, 2’-O-Me is methylation of ribose at the 2’ position [59]. It was revealed that by escaping the suppression mediated by IFN-induced proteins with tetratricopeptide repeats (IFIT), 2’-O-Me-modifiedviral RNA disrupts native host antiviral responses [89]. Surprisingly, 2’-O-Me focuses on modifying specific regions of mRNA where the encoded amino acids are immobilized; these amino acids include glutamate, lysine and glutamine [90]. This phenomenon hints at the hypothesis that 2’-O-Me has the potential to affect translation efficiency, which has previously been demonstrated in bacterial mRNA [81].

### Regulatory roles of RNA modifications in pathogenesis

#### Aberrant m^6^A RNA modifications in diseases

In acute myeloid leukaemia (AML), FTO decreases m^6^A abundance on ASB2 and RARA mRNA in several certain subtypes of AML, including t(11q23)/MLL rearrangements, t(15;17)/PML-RARA, FLT3-ITD, and/or NPM1 mutations [41, 108]. Moreover, by constraining YTHDF2-mediated decay, FTO decreases m^6^A frequency on MYC mRNA [109], METTL3 promotes translation of BCL2 and PTEN mRNA by upregulating the m^6^A levels and supports expression of SP1 by binding to the unique region with the help of the transcription factor CEBPZ [110, 111], and METTL14 enhances mRNA expression of MYB and MYC [112]. All pathological pathways contribute to carcinogenesis in AML. According to the datasets from The Cancer Genome Atlas, nearly 10.5% of AML patients carry copy number variations (CNVs) of ALKBH5, which predicts poor prognosis and p53 mutations [113].

In gastric cancer (GC), METTL3 can cause m^6^A to accumulate on HDGF mRNA, which indicates proliferation and poor prognosis and enhances the stability of zinc finger MYM-type containing 1 (ZMYM1) mRNA so that it accelerates epithelial-mesenchymal transition (EMT) and metastasis [114, 115]. However, METTL3 can also reduce m^6^A on SEC62 with the help of MiR-4429 [116]. In hepatocellular carcinoma (HCC), METTL3 enhances the degradation of m^6^A-containing SOCS2 mRNA together with YTHDF2 [117]. Additionally, YTHDF2 supresses ERK/MAPK signalling cascades and cell proliferation by destabilizing the EGFR mRNA [118]. Regarding clinical diagnosis, downregulated METTL14 is detected in HCC patients, and the level of expression in metastatic HCC is further decreased [119]. In pancreatic cancer, m^6^A and METTL3 protein and mRNA levels were much higher in tumour specimens than in para-cancerous specimens [120]. Meanwhile, upregulation of YTHDF2 destabilizes YAP mRNA by initiating the AKT/GSK3β/cyclin D1 pathway, which promotes proliferation and inhibits the migration of pancreatic cancer [121].

In lung cancer, METTL3 enhances the translation of EGFR and TAZ mRNA [56]. Furthermore, SUMOylated METTL3 promotes non-small-cell lung cancer (NSCLC) by diminishing the amount of m^6^A [122]. Moreover, YTHDF2 enhances the translation of 6-phosphogluconate dehydrogenase (6PGD) mRNA by binding to a given region in lung cancer cells [123]. Additionally, FTO is overexpressed in human NSCLC tissues and stimulates lung cancer by stabilizing and increasing the expression of ubiquitin-specific protease 7 (USP7) [124]. In lung squamous cell carcinoma (LUSC), overexpressed FTO accelerates oncogene MZF1 expression by diminishing m^6^A and stabilizing mRNA as well [125, 126].

For the nervous system, decreased levels of METTL3 or METTL14 determine the diminution of m^6^A on ADAM19 mRNA, which promotes protein expression [127, 128]. Conversely, increased levels of ALKBH5 lead to decreased levels of m^6^A on FOXM1 mRNA and enhance protein expression [129]. Consequently, a high level of ALKBH5 predicts poor prognosis [130]. However, both pathways can contribute to glioblastoma. Subsequently, overexpressed METTL3 recruits HuR to modified SOX2 mRNA and enhances radio-resistance. Playing an oncogenic role in glioblastoma, METTL3 hints at poor prognosis and a potential therapeutic strategy as well [131].

In prostate cancer, reduced YTHDF2 elevates m^6^A contents dramatically, which suppresses proliferation and migration [132]. In bladder cancer, increased METTL3 predicts poor survival because with the help of pri-miR221/222, upregulated METTL3 results in downregulated PTEN and tumorigenesis of cancer [133].

Aberrant m^6^A modification can also lead to carcinomas in the reproductive system. It has been reported that m^6^A on KLF4 and NANOG can be suppressed by the cooperation of ZNF217 and ALKBH5, especially in a HIF-dependent manner, so that it enhances the stability of mRNA and contributes to breast cancer in a hypoxic microenvironment [134, 135]. Increased METTL3 leads to enhancement of m^6^A on hepatitis B X-interacting protein (HBXIP) and proliferation of breast cancer stem cells (BCSCs) [136]. Moreover, elevated FTO leads to downregulated methylation and degradation of BNIP3. It is suggested that FTO enhances the colony formation and metastasis of breast cancer [137]; Nevertheless, in cervical squamous cell carcinoma (CSCC), high expression of FTO and low levels of β-catenin lead to chemoradiotherapy resistance, which hints that FTO is a potential target to increase the chemoradiotherapy sensitivity of CSCC [138]. In endometrial cancer, either mutated METTL14 or reduced METTL3 limits the expression of m^6^A. However, limited m^6^A activates the AKT signalling pathway and stimulates proliferation and tumorigenicity by decreasing the negative AKT regulator PHLPP2 and increasing the positive AKT regulator mTORC2 [139].

Besides the regular cancers with high incidence referenced above, aberrant m^6^A modifications also play roles in sensory organs. The fate of ocular melanoma can be modulated by m^6^A modifications. With the help of YTHDF1, the translation of methylated HINT2 mRNA, a tumour suppressor of ocular melanoma, was significantly accelerated, meaning m^6^A modification obviously inhibits the progression of ocular melanoma. Moreover, investigation of ocular melanoma samples indicated that decreased m^6^A levels were highly associated with poor prognosis [140].

#### Aberrant m^1^A RNA modification in diseases

Physiological functions lead to pathological impacts on diverse diseases. In ovarian and breast cancers, demethylation of m^1^A by ALKBH3 induces increased modified CSF-1 mRNA, which contains m^1^A in the 5’UTR near the translation initiation site. Hence, accumulated ALKBH3 means improved CSF-1 mRNA expression and invasion of cancer cells [141]. Subsequently, ALKBH3, considered the eraser of m^1^A, tightly correlates with the mTOR pathway in gastrointestinal cancer and is attributed to the limited expression of ErbB2 and AKT1S1 after ALKBH3 knockdown; the downstream genes of m^1^A are associated with cell proliferation according to Gene Ontology analysis [142]. Additionally, silencing of ALKBH3 arrests the cell cycle at the G1 phase and contributes to the progression, angiogenesis and invasion of urothelial carcinomas by modulating NADPH oxidase-2-reactive oxygen species (NOX-2-ROX) and TNF-like weak inducer of apoptosis (TWEAK)/Fibroblast growth factor-inducible 14 (Fn14)-VEGF signals [143]. As a classical chemical modification of mRNA, the pathological pathways of m^1^A need to be elucidated.

#### Aberrant m^5^C RNA modification in diseases

Since m^5^C bridges transcription and translation, we propose a hypothesis that m^5^C can also regulate the pathological mechanisms of various diseases. For instance, diminishing NSUN2 leads to decreased levels of translation and an increased tumour initiating population in skin cancer [144]. In breast cancer, NSUN2 is reported to be upregulated as well at the mRNA and protein levels [145]. For patients with urothelial carcinoma of the bladder (UCB), m^5^C-modified 3’UTR in HDGF mRNA can be recognized by YBX1 and activate the oncogene of UCB [78]. m^5^C can also be regarded as a cancer biomarker because the amount of m^5^C RNA modification is increased in circulating tumour cells from patients with lung cancer [146].

#### Aberrant hm^5^C, Ψ, I, U and 2’-O-Me RNA modifications in diseases

Although the amounts of hm^5^C, Ψ, I, U and 2’-O-Me RNA modifications on mRNA are much lower than the three predominant types of modifications, their roles do not change and are vital to human disease. First, Ψ can function as a biomarker for prostate cancer because certain nucleolar RNAs (H/ACA snoRNAs) and the dyskerin (DKC1) protein can upregulate the transformation of U to Ψ and contribute to the advancement to cancer [147]. Regarded as the gene encoding the Ψ synthase, the mutation of DKC1 causes downregulated Ψ and X-linked dyskeratosis congenita (X-DC) [148]. The risk for cancer development is higher in patients with X-DC than those without gene mutation [149]. Besides, H/ACA snoRNAs are limited in acute leukaemia, lymphoma and multiple myeloma [150–152].

Subsequently, edited AZIN1 stimulates a serine to glycine (S/G) conversion in HCC and leads to proliferation and poor prognosis [153, 154]. In HCC and in cervical cancer, increased editing of BLCAP activates the AKT/mTOR signalling pathway or STAT3, which can increase cell proliferation and limit apoptosis [155–158]. In breast cancer, editing of DHFR transcripts at the 3’UTR by ADAR1 stabilizes the mRNA and enhances cell growth. Surprisingly, methotrexate, a chemotherapy agent, prevents cancer cell division by targeting DHFR. It is suggests that downregulated ADAR1 can contribute to methotrexate treatment [159]. In gastric cancer, ADAR2 edits the CDS of PODXL, which induces a histidine to arginine conversion. The relationship between reduced ADAR2 and increased malignancy hints that transcript editing is essential to prevent cancer progression [160]. Additionally, adenosine deaminase RNA-specific B1 (ADARB1), a special type of ADAR, is expressed at low levels in H358 and A549 lung adenocarcinoma (LUAD) cells, which suggests that I might be a potential target in diagnostic and prognostic progression for patients with LUAD [161].

**Fig. 4:**
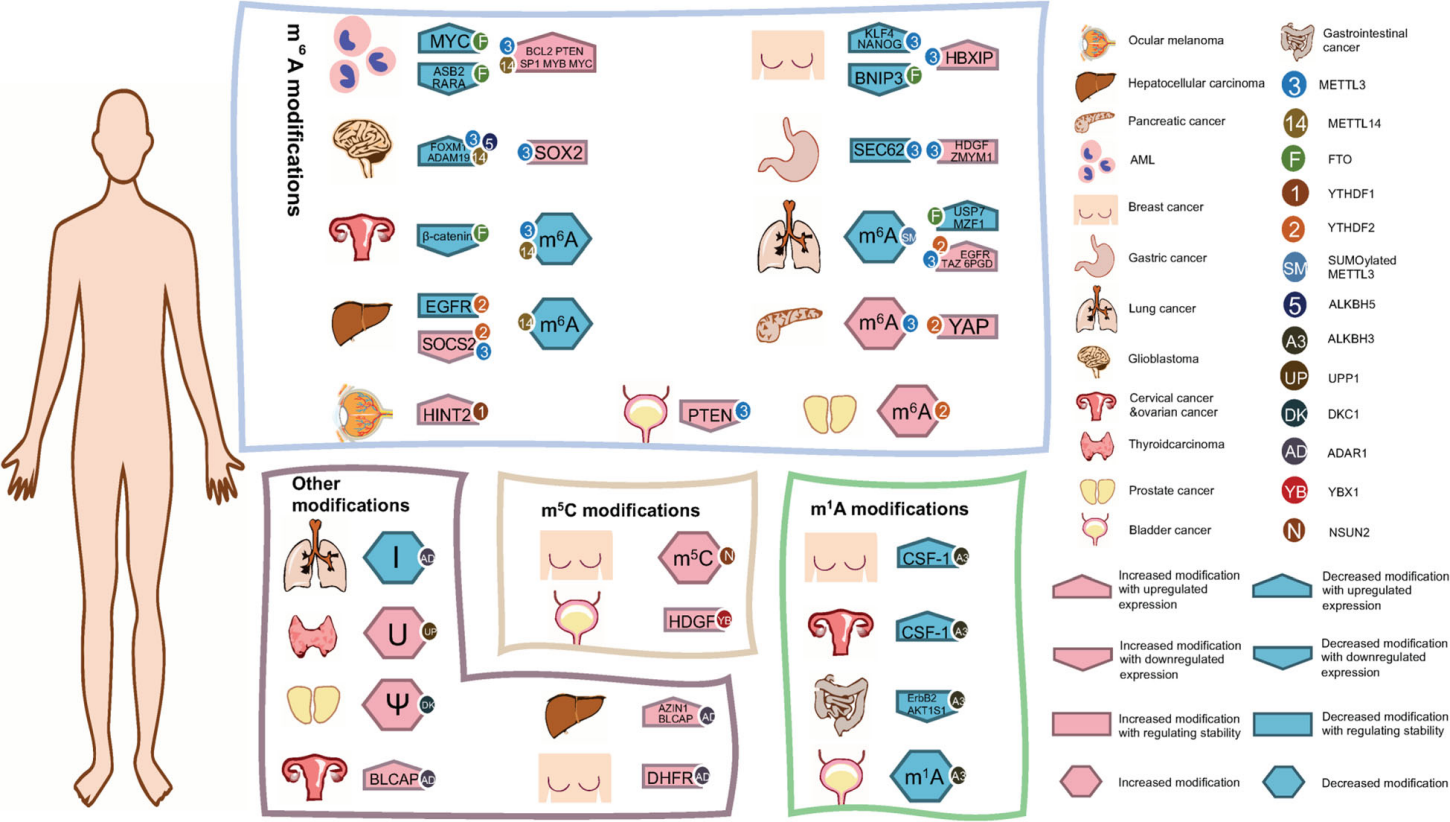
Regulatory roles of RNA modifications in pathogenesis. Applying physiology to pathology, RNA modifications redefine the bridge between transcription and translation and regulate disease pathogenesis. In AML, METTL3 and METTL14 enhance the expression of m^6^A modifications as well as the BCL2, PTEN, SP1, MYB and MYC genes, which lead to tumour progression. Simultaneously, FTO decreases m^6^A abundance on ASB2 and RARA mRNA. In digestive system tumours, aberrant METTL3 leads to aberrant expression of HDGF, ZMYM1, SEC62 and SOCS2, which can regulate cancer cells in the stomach, liver and pancreas, respectively. In lung cancer, METTL3 enhances the translation of EGFR and TAZ, whereas SUMOylated METTL3 promotes NSCLC; aberrant YTHDF2 enhances the expression of 6PGD in lung cancer, and overexpressed FTO stabilizes and accelerates the expression of USF7 and MZF1 as well. In glioblastoma, METTL3, METTL14 and ALKBH5 promote the expression of ADAM19 and FOXM1 and predict poor prognosis. In prostate cancer, aberrant YTHDF2 suppresses proliferation and migration. In bladder cancer, METTL3 reduces the expression of PTEN and tumorigenesis of cancer. In the reproductive system, METTL3 and FTO contribute to the aberrant expression of KLF4, NANOG, HBXIP, BNIP3 and β-catenin, which induce proliferation of breast cancer and chemoradiotherapy resistance of cervical cancer separately. In sensory organs, YTHDF1 accelerates the translation of methylated HINT2 and inhibits the progression of ocular melanoma. Aberrant eraser ALKBH3 reduces m^1^A modifications, leads to aberrant expression of CSF-1, ErbB2 and AKT1S1, and induces the progression of ovarian cancer, breast cancer, gastrointestinal cancer and urothelial cancer. In UCB, YBX1 recognizes m^5^C-modified HDGF mRNA and leads to tumour advancement. Upregulated USUN2 is detected in breast cancer. Ultimately, aberrant ADAR1 edits AZIN1, BLCAP, and DHFR separately, which leads to hepatocellular carcinoma, cervical cancer and breast cancer. Additionally, together with Ψ, I and U, DKC1, ADAR1 and UPP1 can function as biomarkers to indicate prostate cancer progression, LUAD presentation and thyroid carcinoma status

Finally, uridine phosphorylase 1 (UPP1) is another enzyme that can reversibly catalyse the phosphorolysis of uridine to uracil [162, 163]. It has been reported that expression of UPP1 significantly depends on lymph node metastasis and tumour stage and size in patients with thyroid carcinoma [164] (Table 3, Fig. 4).

**Table 3:** Aberrant mRNA modifications in diseases

Modification	Disease	Enzyme	Target	Description	Ref
m^6^A	AML	FTO	ASB2/ RARA	FTO decreases m^6^A abundance on ASB2 and RARA mRNA in certain subtypes of AML and diminishes the amount of protein	[41, 108]
			MYC	FTO decreases m^6^A frequency on MYC mRNA by limiting YTHDF2-mediated RNA decay	[109]
		METTL3	BCL2/ PTEN	METTL3 promotes the translation of BCL2 and PTEN mRNA by upregulating m^6^A levels	[110]
			SP1	METTL3 supports the expression of SP1 by binding to the unique region with the help of the transcription factor CEBPZ	[111]
		METTL 14	MYB/ MYC	METTL14 enhances the expression of MYB and MYC mRNA in AML	[112]
		ALKBH5	N.A.	Approximately 10.5% of AML patients carry CNVs of ALKBH5, which predicts poor prognosis and p53 mutations	[113]
	Gastric cancer	METTL3	HDGF	METTL3 causes m^6^A to accumulate on HDGF mRNA, which indicates proliferation and poor prognosis of gastric cancer	[114]
			ZMYM1	METTL3 enhances the stability of ZMYM1 mRNA to accelerate EMT and metastasis	[115]
			SEC62	METTL3 reduces m^6^A on SEC62 with the help with MiR-4429	[116]
	Hepatic carcinoma	METTL3	SOCS2	METTL3 works with YTHDF2 together to enhance the degradation of SOCS2 m^6^A-containing mRNA, which leads to HCC	[117]
		YTHDF2	EGFR	YTHDF2 suppresses ERK/MAPK signalling cascades and cell proliferation via destabilizing the EGFR mRNA	[118]
		METTL14	N.A.	The expression of METTL14 is decreased in HCC, especially in metastatic HCC	[119]
	Pancreatic cancer	METTL3	N.A.	METTL3 protein, m^6^A abundance and mRNA levels are much higher in tumour specimens than in para-cancerous specimens	[120]
		YTHDF2	YAP	Increased YTHDF2 promotes proliferation and suppresses migration of pancreatic cancer by destabilizing YAP mRNA	[121]
	Lung cancer	METTL3	EGFR/ TAZ	METTL3 enhances the translation of EGFR and TAZ mRNA in lung cancer	[56]
		SUMOylated METTL3	N.A.	SUMOylated METTL3 promotes NSCLC by diminishing the amount of m^6^A	[122]
		YTHDF2	6PGD	YTHDF2 enhances 6PGD mRNA translation by binding to m^6^A sites uniquely in lung cancer cells	[123]
		FTO	USP7	FTO stabilizes and increases the expression of USP7 by reducing m^6^A content	[124]
		FTO	MZF1	Overexpressed FTO accelerates oncogene MZF1 expression by diminishing m6A and stabilizing MZF1 in LUSC	[125, 126]
	Glioblastoma	METTL3/ METTL14	ADAM19	Decreased METTL3 or METTL14 determines the diminution of m^6^A on ADAM19 mRNA, which promotes the expression of protein and contributes to glioblastoma	[127, 128]
		ALKBH5	FOXM1	Increased levels of ALKBH5 lead to decreased levels of m^6^A on FOXM1 mRNA and enhance protein translation, which predicts poor prognosis	[129, 130]
		METTL3	SOX2	Elevated METTL3 stabilizes SOX2 mRNA and enhances radio-resistance of glioblastoma	[131]
	Prostate cancer	YTHDF2	N.A.	Downregulated YTHDF2 suppresses the proliferation and migration of prostate cancer by elevating m^6^A contents	[132]
	Bladder cancer	METTL3	PTEN	With the help of pri-miR221/222, upregulated METTL3 leads to downregulated PTEN and tumorigenesis of cancer	[133]
	Breast cancer	ALKBH5	KLF4/ NANOG	m^6^A on KLF4 and NANOG can be suppressed by the cooperation of ZNF17 and ALKBH5 to promote protein expression and contribute to breast cancer	[134, 135]
		METTL3	HBXIP	Enhanced levels of m^6^A on HBXIP are attributed to increased METTL3 and promote the proliferation of breast cancer stem cells	[136]
		FTO	BNIP3	Elevated FTO leads to decreased expression of BNIP3 and metastasis of breast cancer	[137]
	Cervical cancer	FTO	β-catenin	High expression of FTO and low levels of β-catenin lead to chemoradiotherapy resistance in cervical squamous cell carcinoma	[138]
	Endometrial cancer	METTL14/METTL3	N.A.	Either mutated METTL14 or reduced METTL3 activates the AKT signalling pathway and stimulates proliferation and tumorigenicity by limiting the expression of m^6^A	[139]
	Ocular melanoma	YTHDF1	HINT2	YTHDF1 promotes the translation of methylated HINT2 mRNA and inhibits the progression of ocular melanoma	[140]
m^1^A	Ovarian/Breast cancer	ALKBH3	CSF-1	Accumulated ALKBH3 indicates improved CSF-1 mRNA expression and invasion of cancer cells	[141]
	Gastrointestinal cancer	ALKBH3	ErbB2/ AKT1S1	Aberrant m^1^A modifications regulate gastrointestinal cancer by modulating the mTOR pathway associated with cell proliferation	[142]
	Urothelial carcinoma	ALKBH3	N.A.	ALKBH3 promotes the progression, angiogenesis and invasion of urothelial carcinomas via NOX-2-ROS and TWEAK/Fn14-VEGF signals	[143]
m^5^C	Skin cancer	NSUN2	N.A.	Inactivating NSUN2 prevents protein translation and stimulates the tumour-initiating population of skin cancer	[144]
	Breast cancer	NSUN2	N.A.	NSUN2 is reported to be upregulated at the mRNA and protein levels	[145]
	Urothelial carcinoma	YBX1	HDGF	m^5^C modified 3'UTR in HDGF mRNA can be recognized by YBX1 and activate the advancement of UCB	[78]
	Lung cancer	N.A.	N.A.	M^5^C RNA modification is upregulated in circulating tumour cells from patients with lung cancer	[146]
Ψ	Prostate cancer	DKC1	N.A.	Certain nucleolar RNAs (H/ACA snoRNAs) and DKC1 that transfer U to Ψ contribute to the progression of cancer	[147]
	Haematological malignancies	N.A.	N.A.	H/ACA snoRNAs are limited in acute leukaemia, lymphoma and multiple myeloma	[150–152]
I	Hepatocellular carcinoma	ADAR1	AZIN1	Edited AZIN1 stimulates S/G conversion and induces proliferation and poor prognosis in hepatocellular carcinoma	[153, 154]
		ADAR1	BLCAP	Increased editing of BLCAP accelerates cell proliferation by activating the Akt/mTOR signalling pathway or STAT3	[158]
	Cervical cancer	ADAR1	BLCAP	Increased editing of BLCAP accelerates cell proliferation by activating the Akt/mTOR signalling pathway or STAT3	[157]
	Breast cancer	ADAR1	DHFR	Editing of DHFR by ADAR1 stabilizes mRNA and accelerates cell growth	[159]
	Gastric cancer	ADAR2	PODXL	Downregulated ADAR2 reduces the decoration on PODXL and increases the malignancy of gastric cancer	[160]
	Lung adenocarcinoma	ADARB1	N.A.	ADARB1 has low expression in H358 and A549 lung adenocarcinoma cells	[161]
U	Thyroid carcinoma	UPP1	N.A.	It is reported that the expression of UPP1 significantly depends on lymph node metastasis, tumour stage and size	[164]

### Clinical prospects of RNA modifications

RNA modifications and enzyme complexes exhibit upregulated and downregulated levels of expression in cancers, which means RNA modifications can serve as biomarkers to diagnose diseases in a manner that is helpful and precise. For example, upregulated YTHDF2 is found in pancreatic cancer, increased m^5^C is detected in lung cancer and accumulated Ψ contributes to the advancement of prostate cancer [121, 146, 147]. However, other biomarkers need to be elucidated. Besides biomarkers to diagnose cancers, RNA modifications are also biomarkers to predict patient prognosis. Since they stimulate or inhibit the progression of cancer, RNA modifications have therapeutic potential. 3-deazaadenosine (DAA) interrupts METTL3/14 and inhibits the decoration of m^6^A by obstructing SAH hydrolase [165], SPI1 is considered a potential target for AML because of inhibition of METTL14 [112], and meclofenamic acid (MA), a non-steroidal anti-inflammatory drug, silences FTO by competing for binding sites [166]. Novel targets for treatment of cancer require further investigation.

## Conclusion

In summary, chemical modifications in mRNA are vital for many processes of cell life, such as pre-mRNA splicing, nuclear export, transcript stability and translation initiation. Importantly, RNA modifications play a critical role in driving cell fate in cancer. The importance of the relationship between RNA modification and various diseases cannot be overly emphasized. In this review, we redefined the bridge between transcription and translation and applied it to physiological and pathological processes. To date, we have demonstrated 2 roles of mRNA modifications in transcription. Generally, one type is mRNA modifications that can change the structure of transcripts, and the other is mRNA modifications that can regulate transcription by joining hands with a complex of enzymes, such as METTL3 or NSUN2. Considering that modifications can regulate the fate of diverse diseases, such modifications have the potential to be utilized in targeted therapy. Surely, RNA modifications as well as the related diseases mentioned above are a fraction of those affecting human beings in nature. Thus, these modifications need to be elucidated in the following few years.

## Data Availability

Not applicable

## Abbreviations

2’-O-Me: Ribose-methylation
3’UTR: 3’ untranslated region
5’UTR: 5’ untranslated region
6PGD: 6-phosphogluconate dehydrogenase
Ψ: Pseudouridine
ADAM19: A disintegrin and metallopeptidase domain 19
ADAR: Adenosine deaminases acting on RNA
ADARB1: Adenosine deaminase RNA-specific B1
AKT: AKT serine/threonine kinase
AKT1S1: AKT1 substrate 1
ALKBH5: α-ketoglutarate-dependent dioxygenase alkB homolog 5
ALYREF: Aly/REF export factor
AML: Acute myeloid leukaemia
Arntl: Aryl hydrocarbon receptor nuclear translocator-like
ARE: AU-rich element
ASB2: Ankyrin repeat and SOCS box-containing 2
A-to-I editing: Adenosine-to-inosine RNA editing
AZIN1: Antizyme inhibitor 1
BCL2: B cell leukaemia
BCSC: Breast cancer stem cell
BLCAP: Bladder cancer-associated protein
BNIP3: BCL2 interacting protein 3
CDS: Coding sequence
CEBPZ: CCAAT enhancer binding protein zeta
CNV: Copy number variation
CPE: Cytoplasmic polyadenylation element
CSCC: Cervical squamous cell carcinoma
CSF-1: Colony stimulating factor 1
C-to-U editing: Cytidine-to-uridine RNA editing
DAA: 3-deazaadenosine
DHFR: Dihydrofolate reductase
DKC1: Dyskerin pseudouridine synthase 1
DNMT: DNA methyltransferase homologue
EB: Embryoid body
EGFR: Epidermal growth factor receptor
eIF3: Eukaryotic initiation factor 3
ELAV1: ELAV-like RNA-binding protein 1
EMT: Epithelial-mesenchymal transition
Fasn: Fatty acid synthase
FLT3: Fms-related tyrosine kinase 3
FMR1: Fragile X mental retardation 1
Fn14: Fibroblast growth factor-inducible 14
FOXM1: Forkhead box M1
FTO: Fat mass and obesity-associated protein
GC: Gastric cancer
HBXIP: Hepatitis B X-interacting protein
HCC: Hepatocellular carcinoma
HDF: Human diploid fibroblast
HDGF: Hepatitis B X-interacting protein
HIF: Hypoxia inducible factor
hm^5^C: 5-hydroxymethylcytosine
HNRNP: Heterogeneous nuclear ribonucleoprotein
HuR: Hu antigen R
IFIT: IFN-induced proteins with tetratricopeptide repeats
IGF2BP: Insulin-like growth factor 2 mRNA-binding protein
IL-17A: Interleukin-17A
IRE: Iron-responsive element
KLF4: Kruppel like factor 4
LRPPRC: Leucine-rich pentatricopeptide repeat-containing
LUAD: Lung adenocarcinoma
LUSC: Lung squamous cell carcinoma
m^1^A: N1-methyladenosine
m^6^A: N6-methyladenosine
m^5^C: 5-methylcytosine
MA: Meclofenamic acid
MAPK: Mitogen-activated protein kinase
mESC: Mouse embryonic stem cell
METTL3: Methyltransferase-like 3
METTL14: Methyltransferase-like14
MiR-4429: MicroRNA 4429
MLL: Mixed lineage leukaemia
mRNA: Message RNA
mt-mRNA: Mitochondrial mRNA
mTOR: Mammalian target of rapamycin
MYB: Myeloblastosis oncogene
MYC: Myelocytomatosis oncogene
MZF1: Myeloid zinc finger 1
NANOG: Nanog homeobox
ND5: NADH ubiquinone oxidoreductase core subunit 5
NOX-2-ROX: NADPH oxidase-2-reactive oxygen species
NPC: Neplanocin A
NPM1: Nucleophosmin 1
NSCLC: Non-small-cell lung cancer
NSUN: NOL1/NOP2/SUN domain
NXF1: Nuclear RNA export factor 1
Per2: Period circadian regulator 2
PHLPP2: PH domain and leucine rich repeat protein phosphatase 2
PML: Promyelocytic leukaemia
PODXL: Podocalyxin like
PTEN: Phosphatase and tensin homologue
Q: Queuosine
RA: Rheumatoid arthritis
RARA: Retinoic acid receptor alpha
RBM15: RNA-binding motif protein 15
rRNA: Ribosomal RNA
SAH: S- adenosylhomocysteine
SEC62: SEC62 homologue, preprotein translocation factor
snRNA: Small nuclearRNA
SOCS2: Suppressor of cytokine signalling 2
SOX2: SRY-box transcription factor 2
SP1: Sp1 transcription factor
SPI1: Spi-1 proto-oncogene
SRSF3: Serine and arginine rich splicing factor 3
STAT3: Signal transducer and activator of transcription 3
SUMO: Small ubiquitin-like modifier
T2DM: Type 2 diabetes mellitus
TAZ: Tafazzin
TRMT: tRNA methyltransferase
tRNA: Transfer RNA
TWEAK: TNF-like weak inducer of apoptosis
U2AF: U2 auxiliary factor
UCB: Urothelial carcinoma of the bladder
UPP1: Uridine phosphorylase 1
USP7: ubiquitin-specific protease 7
VEGF: Vascular endothelial growth factor
WTAP: Wilms tumour 1-associated protein
X-DC: X-linked dyskeratosis congenita
YAP: Yes-associated protein
YBX1: Y-Box binding protein 1
YTH: YT521-B homology
ZC3H13: Zinc finger CCCH-type containing 13
ZMYM1: Zinc finger MYM-type containing 1
ZNF217: Zinc finger protein 217
